# Magnesium Can Decrease Postoperative Physiological Ileus and Postoperative Pain in Major non Laparoscopic Gastrointestinal Surgeries: A Randomized Controlled Trial

**DOI:** 10.5812/aapm.12750

**Published:** 2013-12-06

**Authors:** Reza Shariat Moharari, Majid Motalebi, Atabak Najafi, Mohammad Mahdi Zamani, Farsad Imani, Farhad Etezadi, Pejman Pourfakhr, Mohammad Reza Khajavi

**Affiliations:** 1 Department of Anesthesiology and Critical Care, Tehran University of Medical Sciences, Tehran, Iran; 2 Department of Anesthesiology and Critical Care, Iran University of Medical Sciences, Tehran, Iran

**Keywords:** Analgesics, Ileus, Magnesium Sulphate, Pain, Postoperative Period, Vascular Resistance

## Abstract

**Background:**

Magnesium is an antagonist of (N-methyl D-Aspartate) NMDA receptor and its related canals, and may affect perceived pain.

**Objectives:**

The aim of this study was to evaluate the impact of intravenous magnesium on the hemodynamic parameters, analgesic consumption and ileus.

**Patients and Methods:**

A randomized, double blind, placebo controlled study was performed. Thirty two patients of ASA I or II, scheduled for major gastrointestinal (GI) surgery, were divided into magnesium and control groups. Magnesium group received a bolus of 40 mg/kg of magnesium sulphate, followed by a continuous perfusion of 10 mg/kg/h for the intraoperative hours. Postoperative analgesia was ensured by Morphine patient–controlled analgesia (PCA). The patients were evaluated by Intraoperative hemodynamic parameters, the postoperative pain by numeral rating scale (NRS), and the total dose of intraoperative and postoperative analgesic consumption. Postoperative hemodynamic, respiratory parameters, physiological gastrointestinal obstruction (ileus), and side effects were also recorded.

**Results:**

The study included 14 males and 18 females. Age range of patients was 17 to 55 years old. The average age in the magnesium group was 41.33 ± 10.06 years and45.13 ± 11.74 years in control group. Mean arterial pressure (MAP) of magnesium group decreased during the operation but increased in control group (P < 0.001), and systemic vascular resistance (SVR) of magnesium group decreased during the operation also (P < 0.02) but increased in control group. Postoperative cumulative Morphine consumption in magnesium group, was significantly in lower level (P = 0.026). For NRS, severe pain was significantly lower, in magnesium group, at all intervals of postoperative evaluations, but moderate and mild pain were not lower significantly. Duration of postoperative ileus was 2.3 ± 0.5 days in magnesium group, and 4.2 ± 0.6 days in control group (P = 0.01).

**Conclusions:**

Intravenous magnesium reduces postoperative ileus, postoperative severe pain and intra/post operative analgesic requirements in patients after major GI surgery. No side effects of magnesium in these doses were seen, so it seems to be beneficial along with routine general anesthesia in major GI surgeries.

## 1. Background

Magnesium is an antagonist of N-methyl D-Aspartate (NMDA) receptor and its related canals, which may affect perceived pain, and reduce postoperative pain. Intravenous magnesium was introduced for an antiarrhythmic treatment, as well as for the prevention or treatment of seizures in preeclampsia. Magnesium has many physiological activities, and also has the effects of calcium channel blocking. Calcium channel blocking agents have the potential for similar effects of Morphine, in patients with chronic pain (1, 2).

Magnesium sulfate inhibits catecholamine release from the adrenal medulla and peripheral nerve endings, and also blocks catecholamine receptors directly. So magnesium causes sympathetic block and indirectly causes dilated blood vessels, and thereby reduces blood pressure (BP) as an antiarrhythmic effect on heart muscles (3).

The mechanism of action is not clear, but its interfere with calcium channels and NMDA receptors seems to play an important role in this context. In the first place, the effect of magnesium on acute pain was confirmed in a study on rats, and the blockade of calcium channel was seen in this pain relief process (4). In the human body, calcium channel blockers can increase the analgesic effects of opioids in patients with cancer, who are treated chronically with Morphine (5). Secondly, the analgesic effect of magnesium is due to the effect of magnesium on NMDA receptor, NMDA is an amino acid receptor which has excitatory synaptic transmission effects. NMDA has positive modulatory sites (NMDA binding sites) for amino acids such as glutamate, and the negative modulatory site (phencyclidine binding site) for agents such as ketamine or magnesium. Furthermore, it is coupled with the ion channels such as K^+^ and Ca^+^. Magnesium causes a voltage-dependent block of NMDA receptors (6).

In rats, activation of NMDA receptors in the central sensitization process occurs which determines the mode of sensitization of pain after injury (7). Therefore NMDA receptor antagonists may play a role in the prevention and treatment of intra and postoperative pain. In addition, intravenous magnesium increases the effect of other anesthetic drugs, including inhalation drugs (8), and intravenous ones (9). Based on this data, we concluded that magnesium is an NMDA receptor antagonist and a physiologic calcium channel blocker, so it has analgesic properties in acute and chronic pain conditions. In a clinical study, Magnesium administration significantly reduced the rate of postoperative Morphine need in women undergone surgery on the lower abdomen (abdominal hysterectomy) (10).

Postoperative pain affects the patients’ operative outcome (11) and since 1990, the effect of magnesium on postoperative pain and its effect on postoperative opioid consumption, has been studied on gynecologic surgery, ophthalmic surgery, arthroscopic, and lumbar interventions.

Levaux et al. (12) studied 24 patients undergoing major lumbar surgery. In this study, intervention group received 50 mg/kg bolus of intravenous magnesium sulfate. This study revealed that bolus dose reduces postoperative analgesic consumption and makes greater satisfaction and better sleep at first 24 hours in patients undergoing major orthopedic surgery of the spine (12). A few similar studies administered just a single bolus dose of magnesium sulphate just before induction of general anesthesia (13).

In another study on knee arthroscopy, patients were received bolus and infusion doses of magnesium and were assessed for 4 hours after the operation, which the magnesium group received a significantly lower amount of fentanyl, intraoperatively, and postoperatively (2).

Physiologically obstruction (ileus), as a main complication of gastrointestinal surgery, is associated by several causes such as hypomagnesemia (14). Ileus affects hospitalization time and may be minified using intravenous magnesium, and as our knowledge, there are no studies investigating the effect of intravenous magnesium on postoperative ileus time.

## 2. Objectives

The aim of this study was to investigate the effect of intravenous magnesium administration on postoperative pain, hemodynamic and respiratory parameters, following major Gastrointestinal (GI) surgeries.

## 3. Patients and Methods

The study design was a randomized, double blind placebo-controlled clinical trial. Thirty two patients, referred to operation room of Sina hospital (a referral and educational hospital) in Tehran, Iran (from June 2012 to August 2012) were recruited. To prevent bias, the study was designed double blinded and none of the patients and their parents knew about their syringe content, as well as the anesthesiologist who injected anesthetic drugs and filled checklists. One of the authors was aware of the number of cases and grouping, and also the syringe content, and this author delivered syringes to anesthesiologist.

This study was approved by the regional ethics committee of Sina Hospital. Informed consent form was obtained from each patients/guardians.

Patients with age range of 18 to 55 years old, an American Society of Anesthesiologists (ASA) physical status of I or II, and the indication of laparotomy for major GI surgery with a duration of 1 to 3 hours, were included in this study. Exclusion criteria were noncompensated liver failure, renal failure (GFR < 60), heart failure (EF < 45%), any kinds of heart block, any kinds of heart arrhythmia, confirmed hypertension, diabetes, obesity (BMI > 30), neurologic disorders, alcohol and any substances abuse, pregnancy, history of sensitiveness to anesthetic agents, sensitiveness to magnesium compounds and any recent consumption of calcium channel blockers or magnesium compounds. Patients were randomly assigned to magnesium group (n = 16), and control group (n = 16) using a computer-generated randomization list. Routine monitoring of electrocardiogram (ECG), pulse oximetry, and noninvasive blood pressure (NIBP) were conducted prior to induction. Hemodynamic indices, including cardiac output (CO), stroke volume (SV), and systemic vascular resistance (SVR) were monitored by Cardiac output monitoring device (CardioQ-ODM) which is an Oesophageal Doppler Monitor. This advanced device can measure blood flow in the central circulation and is very sensitive to small changes of flow, and also detects the rapid changes of the volume of blood stream. Cardio Q-ODM prevents the reduction of oxygen delivery to the tissues, and also is so easy to be used for intraoperatively.

In the operating room, 8-12 mL/kg/h isotonic saline infusion (N/S) was started and continued during the operation for all patients.

Magnesium group, received 40 mg/kg of magnesium sulphate infusion in 100cc N/S, during 15 min before the induction as the bolus dose, and followed by a continuous infusion of 10 mg/kg/h for the intraoperative hours. The control group received the same volume of an isotonic saline solution.

After at least 2 min of pre-oxygenation, all patients were intubated after administration of fentanyl 2 mcg/kg, Propofol 2 mg/kg, and Atracurium 0.5 mg/kg. During the operation, isoflurane was adjusted to maintain bispectral score (BIS) between 45 and 60 (If the BIS is more than 10 sec outside the scope, isoflurane dosage was changed).

Repeated doses of fentanyl (0.75 mcg/kg) were administered at more than 20% of baseline of heart rate (HR) or mean arterial pressure (MAP).

Baselines of MAP and HR were measured according to the anesthesia clinic examination and examination prior to induction, in the operating room.

All patients got double-lumen central venous catheter, after intubation, and CO, SV and SVR were measured after 30 min of intubation and every next 30 min, during the operation called T1 to T6.

MAP and HR were recorded, before induction of anesthesia, before intubation, immediately after intubation, 5 and 15 min after intubation, and every next 15 min as T1 to T15.

After finishing the operation, postoperative pain was assessed by the patients in the post anesthetic care unit (PACU), with numeric rating scale (NRS), an 11-point scale, divided between zero = no pain to 10 = the worst imaginable pain. Pain severity was reported as none (0), mild (1-4), moderate (5-7), and severe (8-10). At the first 24 hour of postoperative, pain and the analgesic consumption were assessed during the first, second, 4, 12 and 24 hours after the operation. Morphine sulphate was administered as the postoperative analgesic, for all patients, by Morphine patient–controlled analgesia (PCA), containing 2 mg/dose Morphine, with a 15 min lockout interval and no background infusion.

Respiratory rate (RR), HR, MAP, and if there were any side effects of magnesium, were recorded at 15min, 30min, 45min, 1 hour, 2 hours, and 4 hours after the operation. The side effects include respiratory depression, loss of patellar reflex, and urine output < 100 mL/4h.

Duration of physiological gastrointestinal obstruction (ileus) was evaluated after discharge, based on the time of first oral intake tolerance time (oral fluid intake) in medical records.

We determined a sample size of 16 in each group to be sufficient to detect a difference of SVR, estimating a standard deviation of 100 Dyn, a power of 95%, and a significance level of 5%.

The Statistical Package of Social Science version 16.0 (SPSS, Chicago, Illinois, USA) was used for data analysis. Statistical significance was noted for P value of ≤ 0.05. Age, sex, weight, surgery time, intraoperative dosage and renewals of Fentanyl, and the NRS pain score were compared between the two groups by independent sample t-test and chi-square. One-way repeated measures analysis of variance and post hoc Student-Newman-Keuls tests were used to analyze measured NRS and hemodynamic parameters across time within the two groups. Data were expressed as Mean ± SD.

## 4. Results

The study included 16 patients in magnesium group, 6 males (37.5%) and 10 females (62.5%), and of 16 individuals in control group, 8 were male (50%), and 8 were female (50%). There was no significant difference between groups regarding gender (P = 0.34). Age range of patients was 19 to 55 years. The average age in the magnesium group was 41.33 ± 10.06 years and 45.13 ± 11.74 years in the control group. There was no significant difference in age, between groups (P = 0.47). Demographic data, intraoperative analgesic consumption and duration of operation, of both groups are shown in Table 1. Base of mean arterial pressure (MAP) was 101.78 ± 8.97 mmHg, in control group and 96.09 ± 10.76 mmHg in Magnesium group (P = 0.11). Base of heart rate (HR) were respectively 81 ± 9 per min and 85 ± 15 per min, in control group and magnesium group (P = 0.46). 

**Table 1.** Demographic Data for Magnesium and Control Groups ^a^

Parameters	Control (n = 16)	Magnesium (n = 16)	P value
**Age, y**	45.13 ± 11.74	41.33 ± 10.06	0.57
**Sex, male/female**	8/8	6/10	0.36
**Weight, kg**	67.94 ± 6.36	69.36 ± 11.41	0.67
**Intraoperative renewals of fentanyl**	2.19 ± 0.83	1.25 ± 0.68	0.02 ^b^
**Intraoperative doses of fentanyl, µg/kg**	1.48 ± 0.58	1.01 ± 0.61	0.04 ^b^
**Operation Time, min**	173.75 ± 25.17	180.30 ± 23.22	0.75

^a^Data are expressed as (Mean ± SD)

^b^ P < 0.05

**Figure 1.**
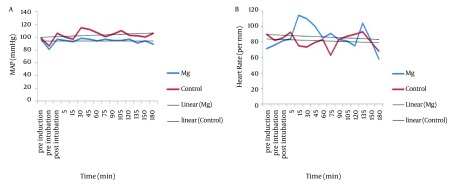
Trends of Intraoperative Mean Arterial Pressure (MAP) (A), and Heart Rate (B), in the Two Groups

**Figure 2.**
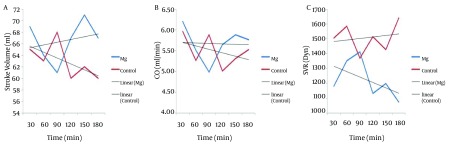
Trends of Intraoperative Stroke Volume (SV) (A), Cardiac Output (CO) (B), and Systemic Vascular Resistance (SVR) (C) in the Two Groups

MAP of magnesium group decreased during the operation but increased in control group (P < 0.001). Intraoperative HR did not show any difference between the two groups (P = 0.19), and did not show any significant decrease in magnesium group (0.39) and control group (0.83) (Figure 1). Trend of intraoperative CO, showed significant decrease in magnesium and control groups (P = 0.001 and P = 0.006 respectively), and also intraoperative SV, showed significant decrease in both magnesium and control groups (P = 0.015 and P = 0.017 respectively) but both of CO and SV did not show any difference between the two groups (P = 0.43 and P = 0.50 respectively) (Figure 2 a and b). Intraoperative SVR, showed significant increase in control group (P = 0.01), but showed significant decrease in magnesium group (P = 0.04), and there was significant difference between the two groups (P < 0.05) (Figure 2 c). 

Postoperative pain severity and analgesic consumption in intervals of first 24 hour were represented in Table 2. For NRS, postoperative pain was significantly lower during the first 24 hour in magnesium group (P = 0.006). 

**Table 2.** Postoperative Pain Severity and Analgesic Consumption

Group	1st Hour		P value	2nd Hour		P value	4th Hour		P value	12th Hour		P value	24th Hour		P value
	C ^a^	Mg ^a^		C	Mg		C	Mg		C	Mg		C	Mg	
**Pain**															
**None**	0	2	NA ^a^	0	3	NA	1	0	NA	2	1	NA	4	1	NA
**Mild**	3	2	NA	4	9	NA	2	4	NA	1	3	NA	3	13	NA
**Moderate**	3	8	NA	8	4	NA	4	7	NA	4	9	NA	8	1	NA
**Severe**	10	4	NA	4	0	NA	9	5	NA	9	3	NA	1	1	NA
**Drug**															
**Morphine, mg/h**	1.81	1.68	0.72	1.07	0.57	0.02 ^*^	1.50	1.75	0.60	2.12	1.36	0.47	0.12	0.07	0.04 ^b^

^a^Abbreviations: C, Control; M, Magnesium; NA, Not available

^b^P < 0.05

**Figure 3.**
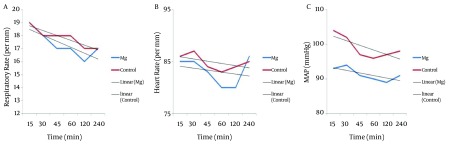
Trends of Postoperative Respiratory Rate (RR) (A), Heart Rate (HR) (B), and Mean Arterial Pressure (MAP) (C) in the Two Groups

between the two groups (P = 0.34 and P = 0.65 respectively). Trend of postoperative RR, showed significant decrease in magnesium and control groups (P = 0.0001 and P = 0.0001 respectively), but postoperative HR did not decrease in magnesium and control groups significantly (P = 0.62 and P = 0.26 respectively). Postoperative MAP did not show any difference between the two groups (P = 0.054) but had significant decrease in both groups (P = 0.046 and P = 0.01 respectively). Trends of postoperative RR, HR and MAP of the two groups were shown in Figure 3. 

## 5. Discussion

The main finding of this study was to reduce the intake of intra and postoperative analgesic and lowering the duration of postoperative ileus, after major non laparoscopic GI surgeries, followed by administration of intra operative magnesium. According to a meta-analysis in 2013, the pain relieving effect of magnesium was examined in a few studies and need to be more evaluated (15).

In Tramer et al. (10) study on abdominal hysterectomy, patients undertook a bolus of 15 mL of 20% magnesium and 2.5 mL/h infusion for 20 hours, and postoperative pain and analgesic consumption were evaluated (10). In the present study, intraoperative and also postoperative pains were evaluated. Another difference between this study and Tramer et al. was the lower dosage for bolus and maintenance of magnesium. In our study, the magnesium group received 40 mg/kg for bolus dose of magnesium sulfate and 10 mg/kg/h, for maintenance in less infusion time, compared with Tramer et al. from the start to the end of operation.

In another study, Telci et al. (9) showed that administration of magnesium sulfate infusion reduced intraoperative anesthetic requirements. Eighty one patients (36 female, 45 male) undergoing elective spinal surgery, were divided into two groups. Intervention group received 30 mg/kg bolus and 10 mg/kg/h infusion during the operation for maintenance. Lower consumed doses of Propofol, remifentanil and vecuronium were identified when magnesium sulfate infusion was performed (9). If the infusion dose of magnesium sulfate was increased, the dosage of anesthetic drugs reduced, but in low dose of 10mg/kg/h, side effects of magnesium were not developed. One of these side effects was dysrhythmias, in form of premature ventricular contraction (PVC), which increased with higher dosage of magnesium, 50 mg/kg compared to 25 mg/kg (16).

Seyhan et al. (17) administered different doses of magnesium sulfate which reduced Propofol consumption and hemodynamics, and postoperative pain was relieved after gynecological surgery (17). Eighty women were divided into four groups. The control group received saline, the 2nd group received only one bolus dose of 40 mg/kg, the next one, bolus plus 10 mg/kg/h infusion, and the fourth group, bolus plus infusion of 20 mg/kg/h magnesium sulfate during 4 hours from the initiation of operation. The results showed that the group receiving 40 mg/kg bolus plus infusion 10 mg/kg/h marked decrease in the use of Propofol, Atracurium and Morphine dose. The increased dose of infusion of magnesium sulfate to 20 mg/kg/h, during and after the operation, improved hemodynamic outcomes, but did not have more effects on reducing anesthetic drugs consumption.

Since the most recommended dietary magnesium sulfate in previous studies, was 40 mg/kg bolus and 10 mg/kg/h infusion during the operation, we analyzed this regimen of administration of magnesium.

Koinig et al. (2) in a study on different types of patients, ASA I and II of the 46 patients who had arthroscopic knee surgery, and general anesthesia was performed in all patients using TIVA with Propofol, Fentanyl and Vecuronium. The intervention group received bolus dose of 40 mg/kg, also received infusion of 10 mg/kg/h. The control group received saline instead of up to 4 hours after the operation; the patients were assessed for pain during and after the operation, and received fentanyl. The results of the group who had received magnesium intra operative and postoperative, a significant (18) study introduced the decreasing effects of major gastrointestinal laparotomy on peri-operative serum level of magnesium (18). Physiologically obstruction (ileus) occurs as a complication of gastrointestinal surgery due to excessive intestinal manipulation, electrolyte imbalance (14), and receiving systemic opioids (19). Surgical stress, as well as causing excessive sympathetic activity could further inhibit intestinal motility (20). By confirmation of the effect of magnesium on sympathetic effects (including the results of several studies such as the present study, on decreasing SVR), magnesium may be effective on postoperative ileus, in major GI surgery. The mechanism of action may be the sympatholysis effect of magnesium and also partially compensation of hypomagnesemia by intravenous magnesium. A few previous studies confirmed that postoperative higher serum magnesium (1.34 ± 0.09) decreases the amount of fentanyl needed (2). In a meta-analysis in 2013, perioperative administration of magnesium sulfate and postoperative pain were evaluated on 25 trial studies, and reported lesser cumulative postoperative morphine consumption by 24.4% reduction and lesser pain score (by NSR method) in the first 24 hour of operation. But based on this quantitative systematic review, the method of administration was not clear yet and so which one of, the bolus, or continuous infusion, or simultaneous bolus and infusion, is the best method of intravenous administration, and the answer needs more investigations, which analyzed with the same pain score method (NSR) and the same postoperative analgesic (Morphine) (15). Of course some new studies would compare oral and intravenous administration of magnesium (21).

We know that MAP is determined by CO, SVR, and central venous pressure (CVP) according to the relationship between flow, pressure and resistance: MAP = (CO × SVR) + CVP, but CVP is usually at or near 0 mmHg, so this formula is often simplified to: MAP ~ CO × SVR; therefore, changes in either CO or SVR would affect MAP. If CO and SVR change reciprocally and proportionately, then MAP would not change. For example, if CO doubles and SVR decreases by one-half, MAP does not change (if CVP = 0). But intravenous magnesium decreased both CO and SVR in this study. Also, the CO decreased in control group, but SVR decreased only in magnesium group, and increased in control group. It means that magnesium sulphate has valuable effects on SVR and by this way decreased MAP during major GI surgeries. Sanchez-Capuchino et al. mM) after intravenous magnesium, versus after saline administration (0.66 ± 0.05 mM) (10), postoperative serum magnesium would be in normal range after 24 hours of intravenous magnesium infusion. Therefore the intravenous magnesium may prevent or reduce the length of large bowel ileus in GI surgery, which can be considered as a new and positive potential of intravenous magnesium (22). Our study confirmed this preventive effect of magnesium on ileus.

We concluded that intravenous magnesium reduces the intraoperative MAP and SVR, and also postoperative severe pain in non-laparoscopic GI surgeries; In particular, the incidence of severe postoperative pain at all 24 postoperative hours was lower in magnesium group. In non-laparoscopic GI surgeries, intravenous magnesium reduces the amount of intra and postoperative analgesia consumption, and also the duration of postoperative ileus, significantly.

No adverse effects of magnesium were seen in our study dosage, so the routine use of these dosage of magnesium, may be safe along with routine general anesthesia drugs.

Further studies are needed to confirm the association between magnesium and gastrointestinal physiological obstruction (ileus), and molecular studies are needed to clarify the possible association between magnesium and the prevention of postoperative ileus in non-laparoscopic GI surgeries.
